# Comparative Study of the Optical and Dielectric Anisotropy of a Difluoroterphenyl Dimer and Trimer Forming Two Nematic Phases

**DOI:** 10.3390/ma17112555

**Published:** 2024-05-25

**Authors:** Evangelia E. Zavvou, Chris Welch, Georg H. Mehl, Alexandros G. Vanakaras, Panagiota K. Karahaliou

**Affiliations:** 1 Department of Physics, University of Patras, 26504 Patras, Greece; pkara@upatras.gr; 2 Department of Chemistry, University of Hull, Hull HU6 7RX, UK; c.welch@hull.ac.uk (C.W.); g.h.mehl@hull.ac.uk (G.H.M.); 3 Department of Materials Science, University of Patras, 26504 Patras, Greece; a.g.vanakaras@upatras.gr

**Keywords:** liquid crystals, liquid crystal oligomers, nematic–nematic transition, birefringence, dielectric anisotropy, dipolar correlations

## Abstract

We present a comparative study of the optical and dielectric anisotropy of a laterally fluorinated liquid crystal dimer and its homologous trimer, both exhibiting two nematic phases. In the high-temperature nematic phase, both oligomers exhibit positive optical anisotropy with similar magnitude, which, however, is lower in comparison with the optical anisotropy of the monomer. In the same temperature range, the dielectric permittivity along and perpendicular to the nematic director, measured on magnetically aligned samples, reveals negative dielectric anisotropy for both oligomers, which saturates as the temperature approaches the N–N phase transition temperature. Comparison of the dielectric anisotropies of the oligomers with the corresponding anisotropy of the monomer indicates a systematic variation of its magnitude with the number of the linked mesogenic units. Results are compared with the corresponding anisotropies of the cyanobiphenyl dimers, the archetypal compounds with two nematic phases, and are discussed in terms of the dipolar structure of the mesogens and the dipolar correlations in their nematic phases.

## 1. Introduction

Liquid crystal dimers consist typically of two rigid mesogenic units linked via a flexible hydrocarbon spacer [1]. Studies on dimeric liquid crystalline systems were primarily motivated by the dramatic dependence of their Isotropic–Nematic transitional properties on the length and the parity of the flexible central spacer [2,3,4,5]. Over the past decade, the elucidation of the structure–property relationship in dimeric systems has become highly topical due to the discovery of spontaneous mirror symmetry breaking in a nematic fluid comprising achiral dimers with an odd number of atoms in the spacer [6,7,8].

The novel, structurally chiral nematic phase typically appears on cooling from the conventional nematic. It preserves the mass density uniformity of nematics and is characterised by a nanoscale helical modulation of the local direction describing the molecular orientational order [7,8,9,10]. This nematic phase, denoted here as N_x_, is interpreted either as twist–bend nematic (N_tb_) [7,8,11,12] due to the resemblance of its structure to the elastically driven micron-scale modulated twist–bend nematic phase, predicted initially by R. B. Meyer [13] and later by Dozov [14], or as polar-twisted nematic (N_pt_) [15,16,17,18] characterised by a polar director roto-translating in the nanoscale. The prime example of a molecular architecture supporting the formation of the N_x_ phase is the CBnCB dimers, where two rod-like mesogenic cores with strong longitudinal dipoles are linked via an odd-membered alkyl spacer [6,11,19,20]. The overall bent molecular shape that is imposed by the parity of the spacer is an essential structural feature for the incidence of the N_x_ phase [21,22], as the even-membered CBnCB homologues with an overall linear shape are typical nematogens [23].

Reasonably, the N_x_ phase was also sought in dimeric architectures deriving from low molar mass nematogens bearing strong lateral dipoles, such as the ortho-difluoro-substituted terphenyls [24], known to exhibit negative dielectric anisotropy. Indeed, the odd-membered homologues of the DTC5Cn series, featuring mesogenic cores with a laterally fluorinated terphenyl group and a terminal pentyl chain, facilitate the formation of the N_x_ [6,7,25,26,27]. The structure–property relationship in difluoroterphenyl-based systems have been explored through a variety of methods, including optical polarising microscopy observations [6,26,28,29], measurements of the elastic constants [7,26,30,31,32] and birefringence [7,26,30,32], modulated differential scanning calorimetry [27], nuclear magnetic resonance [33], polarised FTIR spectroscopy [34,35], circular dichroism [36], freeze-fracture TEM [7,32] and X-ray scattering experiments [10,25,26,27,29,36,37]. The manifestation of the N_x_ phase is also supported by asymmetric fluorinated dimers [38], as well as by binary systems between DTC5Cn dimers with odd members of the DTC5COn family [39].

Beyond dimers, the N_x_ phase has been identified in a variety of oligomeric architectures, as reviewed in Ref [40], and significant efforts have been devoted towards the elucidation of the effect of the number of mesogenic units on the structure–property relationship of higher oligomers [31,37,41]. Concerning the difluoroterphenyl family, the emergence of the N_x_ phase has also been reported in higher oligomers, such as trimers and tetramers [31,39]. Interestingly, comparison of several physical properties between consecutive difluoroterphenyl-based oligomers, such as the optical anisotropy and the bend elastic constant, revealed an alternation with respect to the number of rigid mesogenic units in the oligomer, suggestive of a novel class of odd–even effects at larger length scales [31,37].

The dielectric properties of such oligomeric systems are of special interest since the flexible spacers render the mesogenic cores highly correlated. In the archetypal symmetric CBnCB dimers, the correlations of the dipolar mesogenic groups imposed by the spacer manifest in the dramatic reduction of the dielectric anisotropy, $\Delta \varepsilon =\varepsilon_{∥}-\varepsilon_{⊥}$, in both nematic phases, which is in stark contrast to the classical behaviour of alkyl-cyanobiphenyls (nCBs). According to our recent work, the trends observed in the static dielectric permittivity of symmetric CBnCB dimers with longitudinal dipoles can be quantitatively described considering strong antiparallel orientational dipolar correlations, both intra- and inter-molecular, along the symmetry axes of both nematic phases [42].

In the case of the difluoroterphenyl-based dimeric systems with negative dielectric anisotropy, dielectric studies are relatively scarce owing to the difficulty of establishing a uniform homeotropic alignment even in the high-temperature nematic phase. This usually requires the implementation of sophisticated protocols for the treatment of glass cells [7,30,31,34], or the use of bare gold electrodes, which in the case of DTC5C7, have been shown to promote the spontaneous homeotropic alignment of the director [26]. In up-to-date reported studies, the magnitude of the dielectric anisotropy in DTC5Cn dimers increases with the increase of the orientational order on cooling [26,30,32,34], similar to the behaviour of the corresponding monomer MCT5 [43].

**Figure 1:**
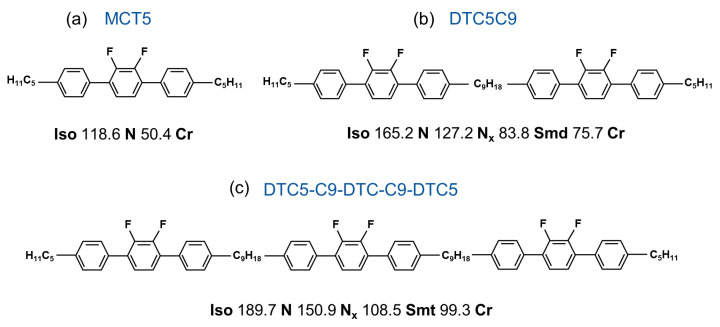
Chemical structures and transition temperatures (cooling) of the studied mesogens: (**a**) the monomer MCT5, (**b**) the dimer DTC5C9 and (**c**) the trimer DTC5-C9-DTC-C9-DTC5.

In this work, we present a comparative study of the birefringence and the static dielectric permittivities of the symmetric odd-membered DTC5C9 dimer and its homologous trimer (DTC5-C9-DTC-C9-DTC5), both bearing nonyl spacers, shown in Figure 1. The positive diamagnetic anisotropy (Δχ>0) of the parent mesogens [26,43] has been exploited for the determination of the dielectric anisotropy in the N phase of the trimer, which has been obtained in planar cells with the aid of a magnetic aligning field. The corresponding monomer MCT5 is also studied as a reference system. The temperature dependence of the components of the static dielectric permittivity, as well as the temperature variation of the optical anisotropy are discussed with respect to the number of the mesogenic units and give valuable insights regarding the relative magnitude of the orientational order, the dipolar correlations and the molecular self-organisation within the nematic phase in N_x_ forming oligomeric systems with negative dielectric anisotropy.

## 2. Materials and Methods

The studied liquid crystalline compounds are the monomeric nematogen 2′,3′-difluoro-4,4″-dipentyl-p-terphenyl (MCT5), the symmetric dimer 1,5-bis(2′,3′-difluoro-4″-pentyl-[1,1′:4′,1″-terphenyl]-4-yl)nonane (DTC5C9) and its homologous trimer 4″,4′′′′′-((2′,3′-difluoro-[1,1′:4′,1″-terphenyl]-4,4″-diyl)bis(nonane-9,1-diyl))bis(2′,3′-difluoro-4-pentyl-1,1′:4′,1″-terphenyl) (DTC5-C9-DTC-C9-DTC5). All three compounds were synthesized at the Department of Chemistry of the University of Hull (UK). Their chemical structure and phase sequences are presented in Figure 1. Transition temperatures were obtained from calorimetric studies on cooling [39].

Phase identification and birefringence measurements were carried out on a Axioskop 40 pol Polarizing Optical Microscope (Carl Zeiss AG, Oberkochen, Germany) equipped with a Linkam LTS420 hotstage (Linkam Scientific Instruments, Salfords, UK) and a Jenoptik ProgRes CT5 camera (Jenoptik AG, Jena, Germany). Birefringence measurements were conducted in planar cells with 5 μm spacing (LCC5 also from Linkam, anti-parallel rubbing) using a Berek compensator (Leitz, Wetzlar, Germany) and monochromatic light of λ=532 nm. The thickness of the empty cells (d=5.00±0.01 μm) was determined using White Light Reflectance Spectroscopy (Theta-Metrisis, Peristeri, Greece).

**Figure 2:**
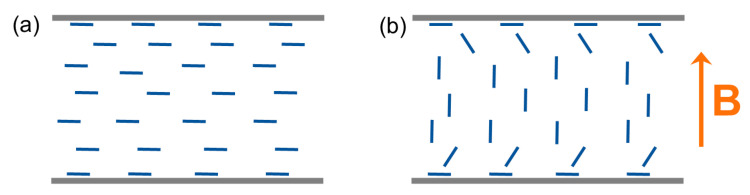
Schematic representation of the director configuration (depicted as line segments) in the sandwich cells used for the dielectric measurements: (**a**) planar alignment of the director in the absence of the magnetic field and (**b**) homeotropic alignment of the director achieved by the application of a sufficiently strong magnetic field (B) across the cell normal.

The $\varepsilon_{⊥}$ and $\varepsilon_{∥}$ components of the static dielectric permittivity in the N phase of all studied systems were extracted from isothermal frequency scans in the range 100 Hz–1 MHz for the monomer and the dimer and 5 kHz–1 MHz for the trimer, employing an Alpha-N Frequency Response Analyser (Novocontrol, Montabaur, Germany). Planar cells with 30 μm spacing and ITO electrodes with sheet resistance of 10 Ω/sq (AWAT, Warsaw, Poland) were filled with the materials in the isotropic phase and placed in a homemade sample holder between the Helmholtz coils of an electromagnet. The capacitance of the empty cells was determined prior to sample preparation. Measurements were performed on cooling from the isotropic phase with various temperature steps. The $\varepsilon_{⊥}$ component of static permittivity was measured in the geometry of Figure 2a, using a $0.2{\;V}_{\mathrm{rms}}$ probe field. For the determination of the $\varepsilon_{∥}$ component, the positive diamagnetic anisotropy Δχ>0 of the studied mesogens allows for the director reorientation through the application of a magnetic field along the cell normal (Figure 2b). Measurements of $\varepsilon_{∥}$ were performed using the maximal magnetic field strength B=1.4 T of our set-up and $0.2{\;V}_{\mathrm{rms}}$. At the transition to the N_x_ phase, we found that the magnetic field can neither induce a homeotropic alignment of the director, nor retain it upon cooling from the N phase in the presence of the field. Therefore, measurements were restricted to the high-temperature N phase. The temperature was controlled using an ITC502S temperature controller (Oxford Instruments, Abingdon, UK), allowing for temperature stabilisation better than ±0.1 °C, while data acquisition and storage were performed with WinDETA v5.66 software, also from Novocontrol.

Capacitance vs. magnetic field curves were also acquired ($0.2{\;V}_{\mathrm{rms}}$) at selected temperatures throughout the N phase of each studied mesogen. The magnetic field strength was continuously varied in steps of 60 mT with a 1 min equilibration interval between measurements. The deviation of $\varepsilon_{∥}$ measured at B=1.4 T from the extrapolated permittivity at the infinite aligning field was determined by plotting $\left(1/\varepsilon^{\prime }\right)$ against $1/\mu_{0}H$, according to the extrapolation method proposed by Clark et al. [44]. In both oligomers, the extrapolated permittivity at an infinite magnetic field was estimated 2% higher than the corresponding values of $\varepsilon_{∥}$ measured at Β=1.4 Τ.

## 3. Results

### 3.1. Optical Anisotropy Studies

**Figure 3:**
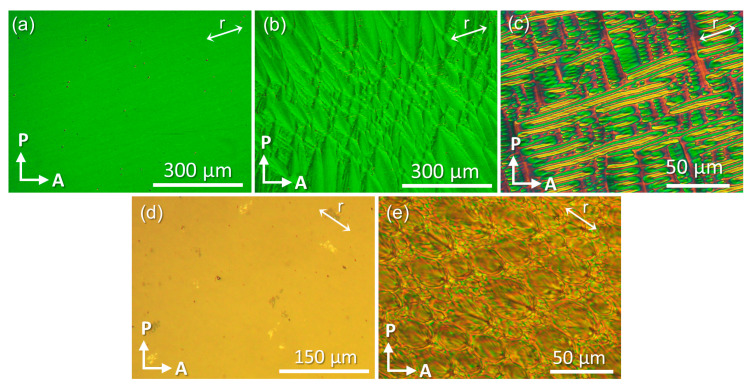
POM textures of the trimer between crossed polarisers. Top row (**a**–**c**) textures in a 5 μm planar cell: (**a**) N phase at 177.0 °C, (**b**) N_x_ phase at 153.8 °C, (**c**) N_x_ phase at 144.9 °C. Bottom row (**d,e**) textures in a 30 μm planar cell: (**d**) N phase at 176.7 °C and (**e**) N_x_ phase at 156.2 °C.

Polarising optical microscopy (POM) observations of the studied compounds in cells treated for planar alignment (5 μm, 30 μm) revealed uniform textures in the N phase of all studied mesogens. Representative examples are shown in Figure 3a,d for the trimer. On further cooling, the N–N_x_ phase transition is marked by the appearance of a blocky texture (Figure 3b), which transforms into the rope-like configurations deep in the N_x_ phase (Figure 3c). In the thicker 30 μm planar cells used for the dielectric experiments, the N phase is homogeneously aligned (Figure 3d), while the textures of the N_x_ phase are dominated by polygonal defects, as shown in Figure 3e for the trimer.

**Figure 4:**
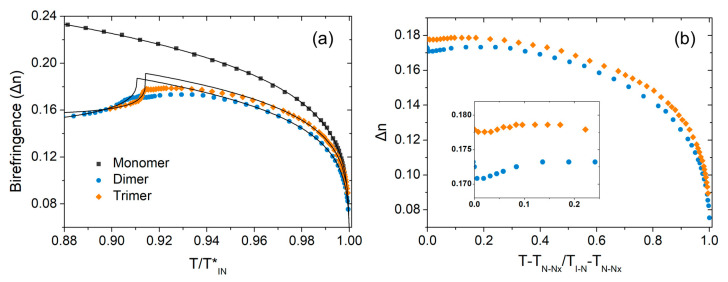
(**a**) Temperature dependence of birefringence of the monomer (squares), the dimer (circles) and the trimer (diamonds), together with the theoretical fittings (solid lines) according to Haller equations. (**b**) Comparison of the temperature dependence of Δn for the dimer and the trimer as a function of the reduced temperature $T_{red}=\left(T-T_{NNx}\right)/\left(T_{IN}-T_{NNx}\right)$.

Figure 4a shows a comparative diagram of the temperature dependence of birefringence of the monomer, dimer and trimer as a function of the reduced temperature, obtained with λ=532 nm. It is evident that the birefringence of the monomer is the highest in magnitude, while the two oligomers exhibit comparable values of birefringence and are lower than that of the monomer. The Haller formula $\Delta n=\Delta n_{0}{\left(1-T/T_{IN}^{*}\right)}^{b}$ [45] allows for the excellent representation of the obtained results for the monomer across the whole range of the N phase, with fitting parameters $\Delta n_{0}=0.348,\;b=0.189$ and $T_{IN}^{*}=393.84\;K$. The results presented here are in agreement with those reported from Cukrov et al. [30] and Saha et al. [37].

In the case of the dimer and trimer, birefringence increases sharply on cooling in the N phase in a similar manner to the monomer. However, at reduced temperatures deeper in the N phase $\left(Τ≲0.96T_{IN}^{*}\right)$, the increasing trend is suspended and a saturation of Δn is observed prior to the N–N_x_ phase transition. This behaviour is similar to that reported for the shorter homologue DTC5C7 [26], as well as to several other N_x_ forming systems [41,42,46,47]. These trends are mainly connected with the temperature variation of the orientational order parameter of the mesogenic groups. In NMR experiments of DTC5C9 [33], as well as in IR absorbance measurements of DTC5Cn series [35], similar non-monotonous trends has been reported for the temperature dependence of the orientational order parameter and are indicative of the formation of short range molecular correlations deep in the N phase.

In Figure 4b, the birefringence of the dimer and the trimer is presented as a function of the reduced temperature, $T_{red}=\left(T-T_{NNx}\right)/\left(T_{IN}-T_{NNx}\right)$. It is evident that the range, where birefringence deviates from the Haller behaviour, is rather similar in both oligomers, a feature that is probably connected to their similar nematic range (~38 °C). Interestingly, stronger pretransitional effects are observed in the dimer when compared to the trimer, as can be seen in the inset of Figure 4b, a feature that is also observed in measurements of a CB6OCB dimer and its homologous trimer reported by Tuchband et al. [41]. In our case, the trimer exhibits a saturation of Δn close to the N–N_x_ transition, while a distinct decrease of Δn is observed for the dimer. These results are in qualitative agreement with the work by Saha et al. [37], where it is also reported that the homologous tetramer exhibits a similar behaviour to the trimer.

At the N–N_x_ phase transition, a small jump in birefringence is observed in both the dimer and trimer (see inset of Figure 4b), that is in accordance with the weakly first order nature of these transitions [31,39]. On further cooling in the N_x_ phase, birefringence decreases due to the progressive deviation (tilt) of the mesogenic units from the helix axis. The obtained results for the dimer and the trimer are fitted using the Haller formula in the N phase, while in the N_x_ phase, the modified Haller equation $\Delta n\left(T\right)=\Delta n_{0}{\left(1-Τ/T_{IN}^{*}\right)}^{b}P_{2}\left(\cos\theta \left(T\right)\right)$ is employed, where $P_{2}\left(x\right)=\left(3x^{2}-1\right)/2$ is the second Legendre polynomial and $\theta \left(T\right)=\alpha^{\prime }{\left(1-Τ/T_{NNx}^{*}\right)}^{b^{\prime }}$ accounts for the temperature dependence of the tilt of the average direction of ordering of mesogenic units with respect to the helix axis [42,48]. Since birefringence deep in the N phase deviates from the typical Haller behaviour, the temperature range used to obtain the Haller fit in N phase was determined based on the R-sqr criterion. The optimal fitting parameters for all studied compounds are listed in Table 1. It should be noted that the evolution of the rope-like configurations deeper in the N_x_ phase inhibits the accuracy of measurements. For this reason, results obtained from extrapolation to absolute zero should be considered with some caution.

In terms of the magnitude of birefringence, the trend $\Delta n_{monomer}>\Delta n_{dimer}≅\Delta n_{trimer}$ is observed at reduced temperatures within the N phase. We note that the obtained trend is in qualitative agreement with the results reported by Saha et al. [37]. In the same study, results for the homologous tetramer are also presented, revealing a novel class of odd–even effects in liquid crystal oligomers with respect to the number of the mesogenic cores, as odd *n*-mers (monomer, trimer) exhibit higher Δn values than even *n*-mers (dimer, tetramer) [37].

Considering that the magnitude and the temperature dependence of the birefringence are mainly determined by the orientational order parameter S of the mesogenic cores with respect to the nematic director, a conclusion that can be drawn is that the magnitude of S in the monomer is the largest between the three, while S values in the dimer and the trimer are of similar magnitude. This trend is also reflected in $\Delta n_{0}$ values extracted through Haller fitting (see Table 1). In all cases, the orientational order parameter is inherently connected with the molecular shape. The overall rod-like shape of the monomer offers better compatibility with the nematic environment when compared to the bent shape of the dimer and the zig-zag shape of the trimer; the latter two considered with the flexible spacers in the all-trans conformation. Taking into account that the ratio of the mesogenic groups to hydrocarbon groups does not change significantly going from the dimer to the trimer and the similar magnitude of Δn, it seems reasonable to argue that the overall orientational distribution of the mesogenic cores with respect to the nematic director is qualitatively similar for both oligomeric structures.

### 3.2. Dielectric Anisotropy Studies

**Figure 5:**
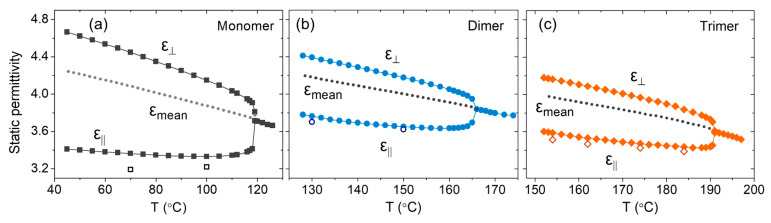
Temperature dependence of static dielectric permittivity (*f* = 22 kHz) in the N phase of the (**a**) monomer, (**b**) dimer and (**c**) trimer. Open symbols represent the calculated infinite field permittivity. Dashed lines correspond to mean permittivity, $\varepsilon_{mean}=\left(2\varepsilon_{⊥}+\varepsilon_{∥}\right)/3,$ and solid lines are guides to the eye.

Dielectric anisotropy studies were conducted in 30 μm planar cells with the aid of a magnetic aligning field. Prior to the dielectric experiments, the homogeneous alignment of the director in the N phase was verified through POM observations (see Figure 3d). The acquired dielectric spectra of all three studied compounds are characterised by a very-low amplitude relaxation mechanism, that appears to shift to lower frequencies on cooling, in both sample alignments. No further analysis was conducted as the frequency of the maximum loss (*f*_max_ = 1/2π*τ*_max_) remained outside of the measuring frequency window in the whole temperature range of measurements. The static dielectric permittivity was determined at f=22 kHz for monomer, dimer and trimer and its temperature dependence is presented in Figure 5a–c, respectively.

The first observation is that the trends for the static dielectric permittivity of the dimer and trimer are similar to those of the analogous monomer. Specifically, in the isotropic phase, the dielectric permittivity increases slowly on cooling assuming values around 3.8 close to the Isotropic–Nematic (I–N) phase transition. At the transition to the N phase, the long-range orientational order results in an increase in the $\varepsilon_{⊥}$ component, a trend that is sustained in the whole range of the N phase. The I–N phase transition is also accompanied by a small jump of $\varepsilon_{∥}$ towards lower values, which upon further cooling, follows a slowly increasing trend. As a result, the dielectric anisotropy of the trimer is weakly negative and its magnitude increases with the increase of the orientational order.

**Figure 6:**
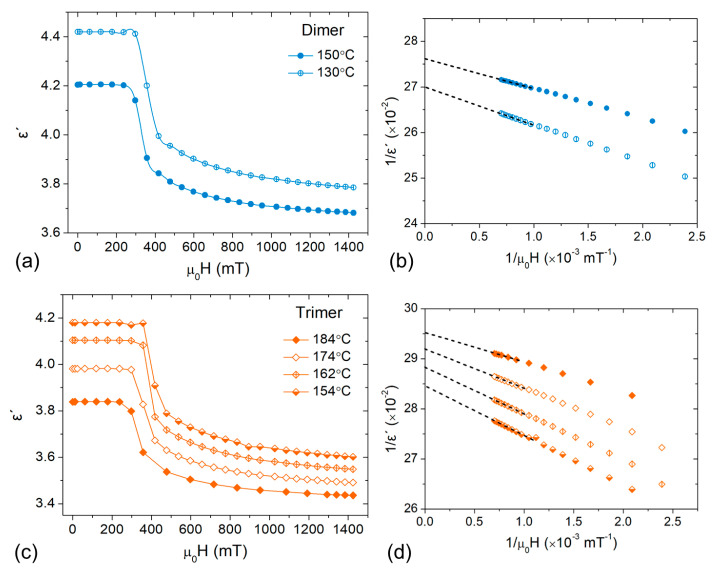
(Left column) Static dielectric permittivity (f = 22 kHz) as a function of the applied magnetic field in selected temperatures within the N phase of the (**a**) dimer and (**c**) trimer. Lines are guides to the eye. (Right column) Determination of the infinite field permittivity of (**b**) the dimer and (**d**) the trimer through the extrapolation of the reciprocal of the dielectric permittivity vs. $1/\mu_{0}H$.

In order to distinguish to which extent the gradual increase of $\varepsilon_{∥}$ on cooling is affected by the increased difficulty of the magnetic field to retain the homeotropic alignment, associated with an increase of the splay elastic constant $K_{11}$ on cooling [26,31,32], the infinite field parallel permittivity was determined through permittivity measurements as a function of the applied magnetic field. The obtained curves are shown in Figure 6a,c for the dimer and trimer, respectively. In all studied compounds, the onset of the Freedericksz transition is sharp and detected around 200–400 mT, being slightly higher in the trimer compared to the dimer, in line with the trend reported for $K_{11}$ values and the increasing polymeric character of the system [31]. Further increase of the magnetic field strength above 1 T allows for saturation of permittivity. For materials with positive diamagnetic anisotropy (Δχ>0), the infinite field parallel permittivity can be evaluated by plotting $\left(1/\varepsilon^{\prime }\right)$ against $\left(1/\mu_{0}H\right)$ and using a least-square linear fitting in the part of the curve, where permittivity saturates [44], shown in the graphs of Figure 6b,d. The calculated infinite field permittivity values are included in the graphs of Figure 5 with open symbols.

**Figure 7:**
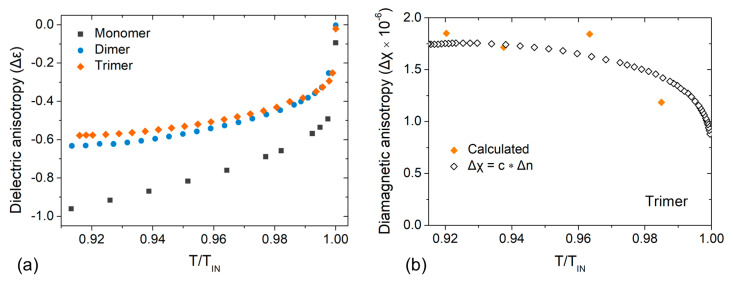
(**a**) Dielectric anisotropy (f = 22 kHz) as a function of the reduced temperature $\left(T/T_{IN}\right)$ for the monomer (squares), dimer (circles) and trimer (diamonds). (**b**) Diamagnetic anisotropy of the trimer calculated (solid diamonds) for selected temperatures using $K_{11}$ values from Ref. [31] and reproduced for the whole nematic range (open diamonds) combining birefringence measurements.

A comparative graph of the temperature dependence of the dielectric anisotropy is presented in Figure 7a as a function of the reduced temperature, $T/T_{IN}$. The absolute values of Δε increase on cooling in all three studied systems. The obtained results for the monomer and the dimer are in good agreement with earlier studies [30,32,43]. However, a saturation of Δε in the dimer and trimer is observed as the N–N_x_ phase transition approaches, in line with the observed trends earlier studies in difluoroterphenyl-based systems [26,30,32]. In terms of the magnitude, it holds that ${\left|\Delta \varepsilon \right|}_{monomer}>{\left|\Delta \varepsilon \right|}_{dimer}≅{\left|\Delta \varepsilon \right|}_{trimer}$. The dielectric anisotropy of the monomer is the highest, similar to the behaviour of the cyanobiphenyl systems, while the dielectric anisotropy of the dimer and the trimer are comparable in magnitude, reflecting once more the similar magnitude of the orientational order parameter. Interestingly, the odd–even effects observed for birefringence are not observed in the dielectric anisotropy, as the magnitude of the dielectric anisotropy tends to decrease with the increase of the number of mesogenic units.

Using the values of the splay elastic constant $K_{11}$ of the trimer reported by Parsouzi et al. [31] and the experimentally determined critical magnetic field $B_{th}$ for the splay Freedericksz transition at similar reduced temperatures, the diamagnetic anisotropy (Δχ) of the trimer can be calculated through the relationship $\Delta \chi =\frac{\pi^{2}}{d^{2}}\frac{\mu_{0}K_{11}}{B_{th}^{2}}$, where $\mu_{0}$ is the magnetic permeability of the vacuum and d is the cell thickness. The calculated values of Δχ as a function of the reduced temperature are presented in Figure 7b with closed symbols. Considering that the temperature dependence of Δχ and Δn are both defined by the temperature dependence of the orientational order parameter, a proportional relationship of the form Δχ=c·Δn can be assumed, with c being a proportionality constant. Using the calculated values of Δχ (closed symbols in Figure 7b) and the measured values of Δn, the proportionality constant $\left(c=9.8\times 10^{-6}\right)$ was estimated and used to calculate the temperature dependence of the diamagnetic anisotropy Δχ(T) of the trimer, shown in Figure 7b with open symbols. In terms of the absolute values, at a reduced temperature $T/T_{IN}\approx 0.94$ in the N phase, the obtained value for the trimer $\left(\Delta \chi =1.7\times 10^{-6}\right)$ is comparable to those reported for the analogous dimers DTC5C7 $\left(\Delta \chi =1.2\times 10^{-6}\right)$ [26] and DTC5C9 $\left(\Delta \chi ≅1.4\times 10^{-6}\right)$ [30], which are also obtained through studies of the Freedericksz transition in a magnetic field.

## 4. Discussion

Revisiting the graphs shown in Figure 5, it is rather intriguing that the components of the static dielectric permittivity of the dimer and trimer exhibit a similar temperature dependence when compared to the monomer, in clear contrast to the strong differentiation between the dielectric behaviour of cyanobiphenyl dimers and their corresponding monomers [19,42]. Moreover, in the case of the studied laterally fluorinated compounds, the mean dielectric permittivity, $\varepsilon_{mean}=\left(2\varepsilon_{⊥}+\varepsilon_{∥}\right)/3$, essentially follows the extrapolated permittivity of the isotropic phase in both oligomeric compounds, as shown in Figure 5 with the dashed lines. This trend along with the absence of any strong variation of $\varepsilon_{mean}$ at the I–N transition reflects that the conformational distribution of the oligomers does not exhibit any significant variation at the transition and varies smoothly throughout the nematic phase.

**Figure 8:**
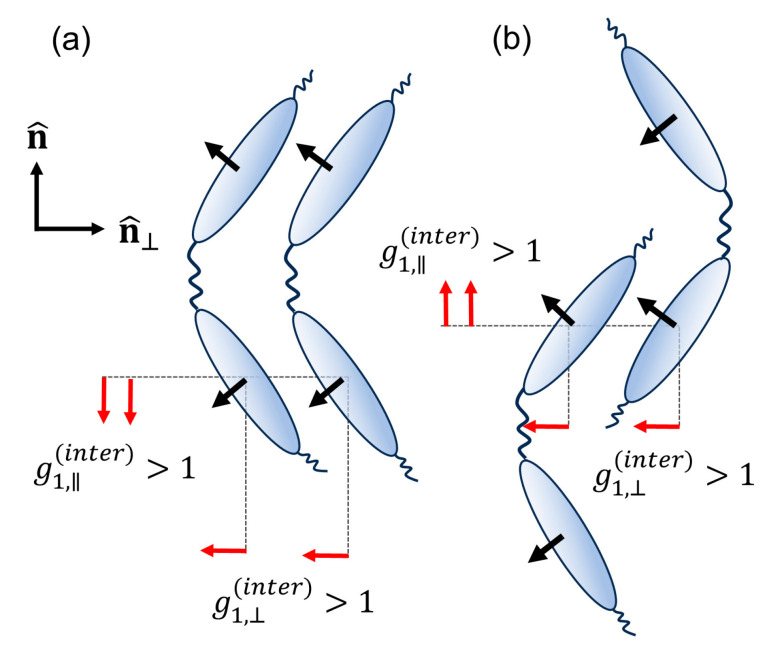
Schematic representation of the inter-molecular dipolar orientational correlations in the nematic phase of difluoroterphenyl-based liquid crystal dimers: (**a**) dimers with coaligned steric dipoles and (**b**) shifted intermolecular arrangement with antiparallel steric dipoles. Black arrows represent the dipole moment of each mesogenic core, while red arrows represent its projections along and perpendicular to the director, $\hat{n}$. In both cases, the inter-molecular dipolar associations promote $g_{1⊥\left(∥\right)}^{\left(inter\right)}>1$, reflecting the parallel and head-to-tail dipolar associations both along and perpendicular to $\hat{n}$. Note here that a simultaneous rotation of the lateral dipoles belonging to neighbouring mesogenic cores (black arrows) by 180° with respect to the core axes leaves $g_{1⊥\left(∥\right)}^{\left(inter\right)}$ unaltered.

The smooth, almost linear, increasing trend of $\varepsilon_{mean}$ with temperature, with relatively similar slopes observed in both the monomer and the oligomers, suggests that the local inter-core dipolar associations do not significantly alter between the different systems. Furthermore, the increase of $\varepsilon_{⊥}$ in the N phase of all studied compounds is indicative of a parallel inter-molecular dipolar association perpendicular to the nematic director. Such correlations promote short-range ferroelectric ordering that remains local since the difluoroterphenyl cores can easily rotate about the axis defined by the aromatic rings, as well as due to the internal torsions of the phenyl rings within the cores. The effects of lateral dipoles have been studied in simulations of hard dipolar spherocylinders, where the formation of super-molecular chains of dipoles with head-to-tail dipolar association was observed [49,50]. Moreover, dielectric studies provided also evidence for the parallel inter-molecular dipolar associations in nematic liquid crystals with strong lateral dipoles [51,52,53]. Considering the aforementioned points, the local structure of the dimeric systems can be visualised according to the scheme depicted in Figure 8, where the head-to-tail dipolar associations are sketched in the case of dimers with coaligned steric dipoles (Figure 8a), as well as for a shifted intermolecular arrangement with antiparallel steric dipoles (Figure 8b). This interpretation would not exclude the formation of intermolecular H···F hydrogen bonding, as reported in Ref. [34].

In the case of the trimer, the optical anisotropy results suggest that the magnitude and temperature dependence of the orientational order parameter are similar to the analogous dimer. Moreover, the common features observed in the static permittivities of both the dimer and the trimer suggest that the dipolar correlations do not substantially vary between the two systems. Thus, it is expected that the conformational statistics for a pair of directly bonded monomers in the trimer will be quite similar to the corresponding conformational statistics of the dimer. Nevertheless, a further description of the inter-molecular dipolar correlations should take into account that the zig-zag shape of the trimer supports different kinds of intermolecular arrangements, as the ones suggested from SAXS experiments [37].

Finally, we would like to compare the trends in the temperature dependence of the optical anisotropy and static dielectric permittivities of difluoroterphenyl N_x_ forming dimers and oligomers with the corresponding findings in the family of CBnCB dimers with positive dielectric anisotropy [42,48,54]. In both families, the main features of the temperature variation of birefringence are qualitatively similar and reflect the temperature variation of the orientational order parameter [33,55]. Moreover, it holds that $\Delta n_{monomer}>\Delta n_{oligomer}$ that is in line with the dominant bent molecular shape of the dimers (or zig-zag shape of the trimer) with respect to rod-like monomers.

Considering the temperature dependence of the dielectric anisotropy, it also holds that ${\left|\Delta \varepsilon \right|}_{monomer}>{\left|\Delta \varepsilon \right|}_{oligomer}$ for both families. However, the trends observed in dimers/oligomers compared to the analogous monomers depend critically on the dipolar structure, as well as on the type of dipolar correlations. In oligomers with lateral dipoles, the relatively smooth variation of the conformational properties along with the parallel inter-molecular dipolar correlations result in an increase of |Δε| on cooling similar to the behaviour of the monomer MCT5. This trend also reflects the key features of the temperature variation of Δn, and consequently of S, both of which exhibit a Haller-like increase close to the I–N transition and saturate on approaching the N_x_ phase. Even though birefringence in CBnCB dimers varies in a similar manner, the temperature dependence of Δε does not follow this trend. In the latter case, the strong longitudinal dipole moments of the mesogenic cores result in significant conformational changes near the I–N transition, which, along with the antiparallel intermolecular dipolar associations, are responsible for the strong reduction of Δε upon cooling.

## 5. Conclusions

In this work, the temperature dependence of the optical and dielectric anisotropy of the laterally fluorinated N_x_ forming liquid crystal dimer (DTC9C5) and the analogous trimer (DTC5-C9-DTC-C9-DTC5) have been studied and compared to those of the corresponding monomer MCT5. The magnitude and the temperature dependence of birefringence in both oligomers are similar, a fact that is indicative of the similar orientational order parameter. Both compounds exhibit rather strong pretransitional effects in the temperature dependence of birefringence on approaching the N–N_x_ transition, which are observed stronger for the dimer than the trimer. With the onset of the N_x_ phase, birefringence decreases in both systems as a result of the helix formation. The dielectric anisotropy in the N phase of the trimer is negative and its magnitude is similar to that of DTC5C9. The temperature dependence of the components of the static permittivity in all studied compounds are suggestive of a similar type of dipolar orientational associations.

## Figures and Tables

**Table 1:** Optimal fitting parameters of the birefringence in N and N_x_ phases of the studied systems.

Compound	$\Delta n_{0}$	b	α’	b’	$T_{IN}^{*}$	$T_{N-Nx}^{*}$
Monomer	0.3480	0.189	--	--	393.84	--
Dimer	0.2843	0.173	0.748	0.1905	440.27	400.91
Trimer	0.2841	0.161	0.590	0.1252	466.10	426.12

## Data Availability

Data are contained within the article.
